# Association of the *NEGR1* rs2815752 with obesity and related traits in Pakistani females

**DOI:** 10.1080/03009734.2020.1756996

**Published:** 2020-05-18

**Authors:** Sobia Rana, Maha Mobin

**Affiliations:** Molecular Biology and Human Genetics Laboratory, Dr. Panjwani Center for Molecular Medicine and Drug Research (PCMD), International Center for Chemical and Biological Sciences (ICCBS), University of Karachi, Karachi, Pakistan

**Keywords:** Genetic association, NEGR1 rs2815752, neuronal growth regulator 1, obesity, overweight, single nucleotide polymorphism

## Abstract

**Introduction:** The variant *NEGR1* rs2815752 has recently been linked with obesity in Caucasians. However, a very limited number of studies have examined the association of the *NEGR1* rs2815752 with overweight/obesity in non-Caucasians with no such study ever performed in Pakistani population. Therefore, the present study was undertaken to seek the association of the rs2815752 with overweight, obesity, and related traits in Pakistanis.

**Subjects and methods:** The study involved 112 overweight/control pairs (total 224) and 194 obese/control pairs (total 388). Anthropometric parameters were measured by employing standard procedures. Metabolic parameters were determined by biochemical assays. Behavioral information was collected through a questionnaire. The rs2815752 was genotyped via TaqMan allelic discrimination assay. Regression analyses were employed to analyze the data in SPSS software.

**Results:** The study revealed significant gender-specific association of the rs2815752 with obesity (OR 3.03; CI 1.19–7.72, *p* = 0.020) and some obesity-related anomalous anthropometric traits (weight, BMI, waist circumference, hip circumference, and abdominal and supra-iliac skinfold thicknesses) in females according to dominant model (*h* = 0.023). However, no association of the rs2815752 with obesity-related behavioral and metabolic parameters was observed.

**Conclusion:** The *NEGR1* rs2815752 may be associated with obese phenotype and some of the related anthropometric traits in Pakistani females.

## Introduction

Obesity is a metabolic disorder characterized by a chronic imbalance of energy homeostasis due to high energy intake and low energy expenditure, eventually increasing the fat mass to the extent that it becomes harmful to health (1). Its prevalence has extended to epidemic proportions worldwide over the past ∼50 years. It substantially escalates the risk of various non-communicable diseases that in turn lead to high rates of morbidity, mortality, and healthcare costs. Moreover, it also significantly increases the economic burden due to its association with unemployment, social disadvantages, and reduced socio-economic productivity. Thus, it has become a major health challenge (2). It is a complex and multifactorial malady attributable not solely to multiple developmental, environmental, behavioural, and genetic factors but also to the interactions between these factors (3,4).

Familial aggregation analysis such as twin and adoption studies has provided compelling evidence for the existence of genetic components in determining obesity and body fat distribution (5). Genome-wide association studies (GWAS) have identified many genes that confer a predisposition to weight gain (6). The genes near body mass index (BMI; a measure of obesity)-regulating loci are enriched for expression in the central nervous system (CNS), indicating that BMI is primarily regulated by processes such as hypothalamic control of eating behaviour, while genes for fat distribution are enriched in local fat depots, indicating that fat distribution is mainly regulated in the adipose tissue itself (7).

Neuronal growth regulator 1 (*NEGR1*) has recently been identified as a new locus responsible for human body weight control in three independent GWAS (8–10). The *NEGR1* gene is positioned on chromosome 1p31.1 and is mainly expressed in the hypothalamus (a crucial center for energy balance and regulation of food intake). However, it is also expressed in subcutaneous adipose tissue (SAT), heart, and skeletal muscles (11). Differential co-expression analysis of obesity-associated networks in human subcutaneous adipose tissue revealed differential expression of the *NEGR1* gene between normal-weight and obese siblings with identification of NEGR1 as a central hub in an obesity-related transcript network (12). However, *in vivo* studies have shown varying results for correlation between the expression level of *NEGR1* and manifestation of obesity, perhaps owing to highly complex regulatory processes of energy homeostasis. The functional inactivation of *NEGR1* in mice led to a small but steady decrease in body mass. In addition, mice carrying a loss-of-function mutation (*NEGR1*-I87N) showed decreased food intake but normal energy expenditure, thereby supporting the positive correlation between *NEGR1* expression and obesity (13). However, another *in vivo* study involving the use of an adeno-associated virus revealed that the decreased expression of *NEGR1* in periventricular hypothalamic areas of rats resulted in escalated bodyweight presumably via increased food intake, but *NEGR1* overexpression did not affect body weight or food intake (14).

Recently, *NEGR1* has been found to be involved in intracellular cholesterol homeostasis, which signifies its non-CNS function associated with human obesity (15). More recently, *NEGR1* deficiency in mice has been found to induce abnormal fat deposition in various peripheral tissues, particularly fat and liver tissue cells, again indicating that the obesity-related function of *NEGR1* is not restricted to the CNS but extends to peripheral tissues as well (16). The rs2815752 variant located upstream of the *NEGR1* gene involving G > A transition has been reported to have one of the biggest effect sizes on BMI values in GWAS conducted on Caucasians (8–10). A very limited number of studies have examined the association of the *NEGR1* rs2815752 with overweight/obesity in non-Caucasians, with no such study ever performed in Pakistani population. Genetic architecture may considerably differ across populations. Thus, genetic association studies should be performed in diverse populations, particularly in under-represented populations, to have a better understanding of genetic variants associated with a trait (17). Pakistan has recently attained a 9th position among 188 countries in terms of obesity, and nearly one-third of the country’s population is overweight/obese (18). Moreover, the Pakistani population, owing to its distinctive features including large size, consanguinity, ethnic diversity, large pedigrees, and rapid nutritional transition, offers many benefits in expounding the pathophysiology of obesity (19). By keeping in view the entire aforesaid scenario, the current study was carried out to examine the association of the *NEGR1* rs2815752 with overweight/obesity and obesity-related multiple traits in a sample of the Pakistani population.

## Materials and methods

### Subjects and ethics

The study was executed at the International Center for Chemical and Biological Sciences (ICCBS), University of Karachi, Pakistan, and after approval from the Independent Ethics Committee (IEC) of the institute and Advanced Studies and Research Board (ASRB) of the University. Moreover, the study was performed in accordance with the ethical standards of the Helsinki declaration. The study encompassed a total of 612 human subjects of both sexes, whose ages ranged from 12 to 63 years. Written informed consent was received from all participants before their participation in the study. The study was based on a case-control design. The total study population of 612 individuals included 112 overweight/control pairs (total 224) that had been age (±5 years)- and sex-matched, and 194 obese/control pairs (total 388), age (±5 years)- and sex-matched. For inclusion of overweight (OW), obese (OB), and normal weight (NW) subjects in the study, BMI cut-off values set by the World Health Organisation (≥18.5 kg/m^2^ for normal weight, ≥25 kg/m^2^ for overweight, and ≥30 kg/m^2^ for obesity) were used for adults (≥20-years-old), whereas BMI growth charts (5th to 84th percentile for normal weight, 85th to 94th percentile for overweight, and ≥95th percentile for obesity) set by Centre for Disease Control and Prevention were used for children and adolescents (<20-years-old). The exclusion criteria were: a history of endocrine disorders like pituitary dysfunction, Cushing’s syndrome, and hypothyroidism; and also a history of medication such as tricyclic antidepressants, anticonvulsants, phenothiazine, and steroids. The technique of simple random sampling without replacement was employed for the recruitment of subjects. In the sampling procedure, each study subject (obese/overweight/control) was recruited randomly from the general population of Karachi in such a way that every individual had an equal chance of being selected, based on the inclusion/exclusion criteria, and no subject was included more than once in the sample. Karachi is a cosmopolitan city with a population of varied ethnic backgrounds from all over Pakistan. Thus, the participants of the study were from diverse ethnic backgrounds including Urdu-speaking, Punjabi, Pashtun, Sindhi, Balochi, and other ethnicities.

### Collection of behavioural data

Obesity-related behavioural information including eating timings, diet unconsciousness, and tendency towards fat-dense food was collected from each participant through a questionnaire.

### Collection of anthropometric data

Anthropometric measurements were performed following standard procedures. Height in centimetres and weight in kilograms were measured using a stadiometer (Seca 214, Germany) and a mechanical column scale (Seca 755), respectively. Skin fold thicknesses (SFT) in millimetres were measured at six different body sites including abdomen, sub-scapula, supra-ilium, thigh, biceps, and triceps using a skin-fold calliper (Slim Guide, MI). Waist and hip circumferences in centimetres were measured using a non-stretchable measuring tape. Subsequently, body mass index (BMI), percent body fat (%BF), and waist-to-hip ratio (WHR) were derived using the measurements mentioned above. BMI was calculated as a person’s weight in kilograms (kg) divided by the square of his/her height in metres (m^2^). The %BF was calculated from skin-fold measurements of abdomen, supra-ilium, thigh, and triceps, using specific formulae for males and females:

$$\text{Males }\%\text{BF}=\left(0.\text{29288}\right)\times \left(\text{4}\text{folds}\right)-\left(0.000\text{5}\right)\times {\left(\text{4}\text{folds}\right)}^{\text{2}}+0.15845\times \left(\text{age}\right)-5.76377$$

$$\text{Females }\%\text{BF}=\left(0.29669\right)\times \left(4\text{folds sum}\right)-\left(0.00043\right)\times {\left(4\text{folds sum}\right)}^{2}+0.02963\times \left(\text{age}\right)+1.4072$$

Waist-to-hip ratio (WHR) was calculated as waist circumference (WC) divided by hip circumference (HC).

### Collection of blood sample

A venous blood sample in a volume of 5 ml was drawn from each participant of the study after an overnight fast (8–12 h) with a 5 ml syringe. Out of this 5 ml sample, 2 ml was collected in an EDTA-coated vacutainer tube for subsequent DNA extraction, while 3 ml blood was collected in another vacutainer tube containing gel and clot activator for subsequent serum isolation. The serum was extracted by centrifuging the tube at 4000 rpm for 10 min.

### Collection of metabolic data

Blood pressure (systolic and diastolic) was recorded twice from the right arm of the subjects, using a mercury sphygmomanometer (Certeza medical, CR-2001, Germany) with an accuracy of 1 mmHg. Fasting blood glucose (FBG) was determined by a blood glucose monitoring system (Abbott, Chicago, IL). Fasting serum insulin concentrations were determined by enzyme-linked immunosorbent assay (ELISA) using a commercially available kit (DIA source INS-EASIA Kit, Cat No. KAP1251, Belgium) on a Multiskan™ FC Microplate Photometer (ThermoFisher Scientific, Waltham, MA). Homeostasis model assessment of insulin resistance (HOMA-IR) was calculated from FBG and fasting insulin values using the formula: HOMA-IR = Fasting insulin (µl U/ml) × Fasting glucose (mg/dl)/405. Homeostatic model assessment of insulin sensitivity (HOMA-IS) was calculated as HOMA-IS = 1 ÷ [fasting insulin (mIU/l) × fasting glucose (mmol/l)]. Assays related to lipid profile including total cholesterol, high-density lipoprotein cholesterol (HDL-C), low-density lipoprotein (LDL-C), and triglycerides (TGs) were performed by using enzymatic *in vitro* assay kits (Merck, Darmstadt, Germany) on a Roche Hitachi chemistry analyzer. Very-low-density lipoprotein cholesterol (VLDL-C) was calculated by dividing TG by 5. Cholesterol-to-HDL-C ratio (CHR) was calculated by dividing the concentration of total cholesterol by the concentration of HDL-C. In addition, different anthropometric and metabolic estimations were used to compute various metabolic indices and ratios including visceral adiposity index (VAI) (mmol/l), lipid accumulation product (LAP) (mmol/l), product of triglyceride and glucose (TyG) index, coronary risk index (CRI), atherogenic index (AI), and triglyceride-to-HDL-C ratio (TG/HDL-C). To calculate VAI and LAP, respective gender-specific formulae were used. To calculate VAI and LAP, values of TG and HDL‐C were converted from mg/dl to mmol/l.

Males VAI=[WC÷39.68+(1.88×BMI)]×[TG÷1.03]×[1.31÷HDL-C]

Females  VAI=[WC÷36.58+(1.89×BMI)]×[TG÷0.81]×[1.52÷HDL-C]

Males  LAP=(WC–65)×TG

Females  LAP=(WC–58)×TG

A general formula for both sexes was used to calculate the TyG index:

TyG  index= Ln [Fasting  triglycerides  (mg/dl)×  Fasting  glucose (mg/dl)÷2]

To calculate CRI, TC was divided by HDL-C, whereas to calculate AI, LDL-C was divided by HDL-C. The TG-to-HDL-C ratio was calculated by dividing the values of TG by HDL-C.

### Genomic DNA extraction and SNP genotyping

Extraction and purification of DNA from whole blood was performed using a genomic purification kit (EZ-10 spin column, CAT #BS483, Bio Basic, Markham, Canada) following the manufacturer’s recommended protocol. Genotyping was performed using TaqMan^®^ predesigned SNP Genotyping Assay (Assay ID: C_26668839_10, Cat No. 4351379, ABI, Foster City, CA) and TaqMan^®^ genotyping master mix (Cat No. 4381656, ABI, Foster City, CA) on an Applied Biosystems 7500 real-time polymerase chain reaction machine (ThermoFisher Scientific, Waltham, MA). The thermal cycling conditions included an initial step of polymerase activation at 95 °C for 10 min followed by 50 cycles of two steps: denaturation at 95 °C for 15 s, and annealing and extension at 60 °C for 1 min. Successful genotyping was performed with 99% genotypic call rates and >95% concordance between duplicate samples. After each run, the post-amplification analysis was done using the software of Applied Biosystems 7500. We included two negative controls (No Template Control or NTC) and one positive control (PC) for each genotype, every time the experiment was run. In addition, genotyping of 20% of the samples was repeated to ensure reproducibility.

### Statistical analysis

Statistical analysis of the data was carried out using the software IBM SPSS Statistics Version 21. Genotypic frequencies in cases and controls were calculated via chi-square, whereas allelic frequencies were calculated by direct count. Genotypic frequencies were reported as counts and percentages. Hardy–Weinberg equilibrium (HWE) analysis for both cases and controls was performed by using a web-based calculator to check whether the genotypic frequencies are in HWE. Logistic regression was performed to examine the association of the variant rs2815752 (G > A) with the risk of overweight/obesity while considering four models of inheritance (co-dominant, dominant, over-dominant, recessive). In the case of getting association in more than one model, the degree of dominance index (*h*) was calculated to select the best-fit model. Age and gender-adjusted odds ratio (OR) and 95% confidence intervals (CI) were calculated to determine the risk of overweight/obesity associated with the rs2815752 variant. The association was also investigated separately in mild (overweight) and severe (obese) phenotypes. In addition, analyses were separately conducted in males and females to identify any gender-specific association in the study sample for which only age was adjusted. Ordinal regression was also performed on the total sample population to assess the association of the variant with BMI grades, taking BMI grades 0 through 4 as an outcome or dependent variable and the dominant genotype of the variant as an explanatory or independent variable. BMI grade 0 was taken for ‘normal’, grade 1 for ‘overweight’, grade 2 for ‘class I obesity’, grade 3 for ‘class II obesity’, and grade 4 for ‘class III obesity’. Parameter estimates were then calculated under PLUM command.

Fisher’s exact test was applied to compare categorical variables, and Mann–Whitney *U* test was applied to compare continuous variables between cases and controls. Categorical variables included demographic characteristics and parameters of eating behaviours, while continuous variables included anthropometric and metabolic traits. Logistic regression was employed to assess the variant’s association with obesity-linked categorical traits, whereas linear regression was employed to assess the variant’s association with obesity-linked continuous variables, using the best-fit model of inheritance. BMI was additionally adjusted while testing metabolic parameters. Statistical significance was taken at a two-sided *p*-values <0.05 for all the comparisons. False discovery rate (FDR) correction for multiple comparisons was performed using the Benjamini–Hochberg method. Effect size with 95% CI was determined for all variables.

### Power calculation

The power calculation was performed employing Quanto software. The rs2815752 risk allele ‘A’ frequency (0.64) and the log-additive model were used to calculate power. Our study was found to have a power of 80% for the least detectable OR of 1.4 with a two-sided type 1 error rate of 0.05.

## Results

A comparison between the characteristics of cases and controls is shown in Supplementary Tables 1–4 (available online). Most of the anthropometric and metabolic parameters were found to be significantly abnormal in cases as compared to controls (Supplementary Tables 1 and 3; available online). No significant differences between cases and controls in terms of parental consanguinity were observed. However, a family history of obesity was found to be significantly higher in cases compared to controls. Abnormal eating behaviours like random eating timings and tendency towards fat-dense food were significantly more prevalent in cases as compared to controls. Nevertheless, no significant differences were observed between cases and controls in terms of diet unconsciousness. Demographic features like percentages of ethnicities are also shown between cases and controls (Supplementary Tables 2 and 4; available online).

Genotypic distribution of the *NEGR1* rs2815752 was observed to be in Hardy–Weinberg equilibrium in both cases and controls (Supplementary Table 5; available online). The genotypic frequencies did not differ significantly between overweight/obese cases and normal-weight controls (Supplementary Table 5; available online). A similar observation was seen between obese males and their controls; however, genotypic frequencies were found to differ significantly between obese females and their corresponding controls (Table 1). Thus, the variant rs2815752 appeared to have a significant gender-specific association with obesity (not with overweight) in females. This association was observed in more than one genetic model (co-dominant and dominant) during age-adjusted regression analysis. The subsequent calculation of the *h* index indicated a dominant mode of inheritance. Thus, according to a dominant genetic model, the GA/AA genotype of the *NEGR1* rs2815752 variant increases 3.03-fold the odds of having obesity in females (Table 1). This finding was further strengthened when the association of the variant rs2815752 with BMI grades was assessed by employing ordinal regression. The analysis revealed a significant association between GA/AA genotype and BMI of higher grades. However, after gender stratification, female carriers but not male carriers of the risk genotype (GA/AA) were found to have a significantly greater risk of having higher BMI grades than non-carriers (GG) (Table 2).

**Table 1.** Genotypic frequencies of the *NEGR1* rs2815752 variant among gender-stratified cases (obese) and controls along with assessment of the association of the rs2815752 with obesity.

		Males				Females			
Genetic model	Genotype	Cases, *n* (%)	Controls, *n* (%)	Adjusted OR (95% CI) ^a^	Adjusted *p*-value^a^	Cases, *n* (%)	Controls, *n* (%)	Adjusted OR (95% CI) ^a^	Adjusted *p*-value^a^
Co-dominant	GG	13 (12.15)	10 (9.35)		0.615	7 (8.05)	18 (20.69)		0.042^b^
	GA	45 (42.06)	41 (38.32)			38 (43.68)	36 (41.76)		
	AA	49 (45.79)	56 (52.34)			42 (48.28)	33 (37.93)		
Pair-wise comparison	GG vs GA			0.84 (0.33, 2.13)	0.720			2.65 (0.98, 7.14)	0.054
	GG vs AA			0.67 (0.27, 1.67)	0.394			3.47 (1.28, 9.38)	0.014 ^b^
Dominant	(GA + AA)	94 (87.85)	97 (90.66)	0.75 (0.31, 1.78)	0.509	80 (91.96)	69 (79.69)	3.03 (1.19, 7.72)	0.020 ^b^
	GG	13 (12.15)	10 (9.35)			7 (8.05)	18 (20.69)		
Recessive	AA	49 (45.79)	56 (52.34)	0.77 (0.45, 1.32)	0.342	42 (48.28)	33 (37.93)	1.75 (0.94, 3.25)	0.076
	(GG + GA)	58 (54.21)	51 (47.67)			45 (51.73)	54 (62.45)		
Over-dominant	GA	45 (42.06)	41 (38.32)	1.17 (0.67, 2.02)	0.582	38 (43.68)	36 (41.76)	1.03 (0.56, 1.90)	0.916
	(GG + AA)	62 (57.94)	66 (61.69)			49 (56.33)	51 (58.62)		
Adjusted *h* index = 0.023									
Hardy–Weinberg Equilibrium (HWE):					*p*-value				*p*-value
Cases					0.597				0.689
Controls					0.584				0.177

Data represent genotype counts and percentages in parenthesis in cases and controls. Data also indicate genotypic frequencies in HWE. Association of rs2815752 with obesity in obese cases was tested using chi-square and multinomial regression for co-dominant model and binary logistic regression for other models.

^a^ Analysis was performed with adjustment for age. ^b^Adjusted *p*-value <0.05 were considered statistically significant. Adjusted *h* index was calculated for indication of appropriate mode of inheritance.

AA: mutant homozygous genotype; CI: confidence interval; GA: heterozygous genotype; GG: wild-type homozygous genotype; OR: odds ratio.

**Table 2.** Assessment of the association of the *NEGR1* variant rs2815752 with BMI grades in the sample population using a dominant model of inheritance.

	All (males and females)		Males		Females	
	Adjusted		Adjusted		Adjusted	
Genotype	OR (95% CI)	*p-*value	OR (95% CI)	*p-*value	OR (95% CI)	*p-*value
GA + AA	1.30 (1.01, 1.67)	0.041^a^	1.12 (0.79, 1.59)	0.512	1.59 (1.10, 2.28)	0.013^a^
GG	1		1		1	

Ordinal regression was used to calculate parameter estimates under PLUM command. BMI grade 0 was taken for normal weight, BMI grade 1 for ‘overweight’, BMI grade 2 for ‘class I obesity’, BMI grade 3 for ‘class II obesity’, and BMI grade 4 for ‘class III obesity’.

^a^ *p*-values <0.05 were considered significant.

When the association of the variant rs2815752 with the obesity-related anthropometric, metabolic, and behavioural traits was assessed, the variant was found to be significantly associated with some of the anomalous anthropometric traits (weight, BMI, WC, HC, and abdominal and supra-iliac skin-fold thicknesses) in females only. On the other hand, no association of the variant was seen with anthropometric traits in males (Table 3). Furthermore, the lack of association between the rs2815752 and obesity-related metabolic traits was observed (Table 4). Similarly, we did not find any association of the rs2815752 with aberrant eating behaviours, except a very nominal (*p* values = 0.048) protective association of the rs2815752 with random eating timings in males. However, no such association was observed in females (Table 5).

**Table 3.** Assessment of the association of the rs2815752 with obesity-related anthropometric variables in gender-stratified obese cases and controls.

Variables (unit of measurement)	Dominant model	Males			Females		
		Mean (SD)	Adjusted *β* (95% CI)	Adjusted *p*-value	Mean (SD)	Adjusted *β* (95% CI)	Adjusted *p*-value (corrected)
Weight (kg)	GG	83.57 (21.20)	−0.848 (−11.139, 9.444)	0.871	62.48 (17.43)	13.102 (3.656, 22.547)	0.033^a^
	GA + AA	82.72 (23.93)			75.55 (22.76)		
Height (cm)	GG	171.38 (6.45)	−0.746 (−3.835, 2.342)	0.634	156.63 (6.27)	0.857 (−1.54, 3.228)	0.477
	GA + AA	170.63 (7.15)			157.50 (5.59)		
BMI (kg/m^2^)	GG	28.51 (7.26)	−0.163 (−3.472, 3.146)	0.923	25.43 (6.80)	5.043 (1.398, 8.688)	0.003^a^
	GA + AA	28.35 (7.66)			30.46 (8.97)		
WC (cm)	GG	100.33 (16.68)	0.016 (−7.952, 7.983)	0.997	92.08 (20.22)	10.907 (1.828, 19.987)	0.049^a^
	GA + AA	100.35 (18.84)			102.91 (21.50)		
HC (cm)	GG	107.08 (14.20)	−0.172 (−6.894, 6.550)	0.960	102.00 (12.26)	7.915 (1.623, 14.207)	0.049^a^
	GA + AA	106.91 (15.65)			109.90 (15.16)		
WHR	GG	0.93 (0.07)	−0.000 (−0.030, 0.030)	0.987	0.89 (0.10)	0.036 (−0.006, 0.078)	0.099
	GA + AA	0.93 (0.07)			0.93 (0.10)		
Biceps SFT (mm)	GG	13.91 (8.46)	−1.426 (−5.198, 2.345)	0.457	15.39 (9.15)	4.372 (−0.343, 8.400)	0.060
	GA + AA	12.49 (8.68)			19.75 (9.48)		
Triceps SFT (mm)	GG	25.09 (14.42)	−1.216 (−7.588, 5.156)	0.707	23.18 (8.54)	4.382 (0.196, 8.567)	0.060
	GA + AA	23.87 (14.63)			27.55 (10.04)		
Abdominal SFT (mm)	GG	42.65 (18.48)	−2.687 (11.677, 6.302)	0.556	29.93 (12.57)	8.571 (2.445, 14.697)	0.033^a^
	GA + AA	39.98 (21.24)			38.46 (15.22)		
Supra-iliac SFT (mm)	GG	39.96 (16.91)	−4.700 (−13.001, 3.601)	0.266	21.71 (10.10)	5.338 (0.810, 9.866)	0.049^a^
	GA + AA	35.27 (19.55)			27.02 (10.90)		
Thigh SFT (mm)	GG	37.35 (24.53)	−0.893 (−11.704, 9.918)	0.871	32.72 (12.70)	6.765 (0.712, 12.818)	0.058
	GA + AA	36.45 (24.87)			39.48 (14.38)		
Sub-scapular SFT (mm)	GG	26.61 (12.57)	−0.495 (−6.297, 5.307)	0.867	24.11 (11.52)	5.774 (0.194, 11.354)	0.060
	GA + AA	26.12 (13.43)			29.87 (13.37)		
Forearm (mm)	GG	281.82 (27.97)	−4.182 (−18.783, 10.419)	0.573	241.86 (30.49)	13.995 (−2.116, 30.106)	0.099
	GA + AA	277.61 (33.25)			255.74 (35.66)		
Percentage of body fat	GG	28.21 (10.69)	−0.997 (−5.664, 3.670)	0.674	28.35 (8.88)	3.672 (−0.421, 7.764)	0.099
	GA + AA	27.22 (10.78)			32.00 (9.86)		

Association with anthropometric traits was assessed by linear regression using dominant model of inheritance. The effect sizes (*β*) with 95% confidence intervals and *p*-values were provided after adjustment for age.

^a^ An adjusted and corrected *p*-value <0.05 was considered statistically significant.

BMI: body mass index; CI: confidence interval; HC: hip circumference; SD: standard deviation; SFT: skin fold thickness; WC: waist circumference; WHR: waist-to-hip ratio.

**Table 4.** Assessment of the association of the rs2815752 with obesity-related metabolic variables in gender stratified obese cases and controls.

Variables (unit of measurement)	Dominant model	Males			Females		
		Mean (SD)	Adjusted for age and BMI		Mean (SD)	Adjusted for age and BMI	
			*β* (95% CI)	*p*-value		*β* (95% CI)	*p*-value
Systolic BP (mmHg)	GG	119.57 (11.47)	2.510 (−2.986, 8.006)	0.369	110.72 (12.50)	0.528 (−5.144, 6.200)	0.854
	GA + AA	122.01 (13.60)			116.25 (16.91)		
Diastolic BP (mmHg)	GG	77.39 (9.15)	3.334 (−0.938, 7.607)	0.125	73.60 (8.10)	2.125 (−2.463, 6.712)	0.362
	GA + AA	80.65 (10.61)			77.60 (11.53)		
VAI (mmol/l)	GG	3.65 (1.79)	−0.615 (−1.665, 0.435)	0.249	3.31 (2.35)	0.000 (−0.83, 0.830)	1.00
	GA + AA	3.03 (2.57)			3.60 (1.91)		
LAP (mmol/l)	GG	63.87 (42.92)	−6.770 (−23.242, 9.703)	0..419	44.69 (36.35)	−2.721 (−15.016, 9.574)	0.663
	GA + AA	56.50 (50.32)			60.72 (44.02)		
TyG index	GG	8.91 (0.43)	−0.250 (−0.471, −0.028)	0.027	8.55 (0.46)	−0.053 (−0.244, 0.139)	0.587
	GA + AA	8.66 (0.57)			8.59 (0.48)		
FBG (mg/dl)	GG	102.26 (14.46)	−0.60 (−10.44, 9.24)	0.904	106.40 (19.28)	−4.042 (−12.947, 4.863)	0.372
	GA + AA	101.62 (24.38)			105.38 (21.30)		
Insulin (µl U/ml)	GG	22.01 (9.12)	1.99 (−4.76, 8.73)	0.562	23.57 (13.57)	−1.673 (−6.935, 3.589)	0.531
	GA + AA	23.84 (17.94)			25.12 (13.12)		
HOMA-IR	GG	5.61 (2.64)	0.48 (−1.57, 2.52)	0.642	6.23 (3.55)	−0.546 (−2.128, 1.035)	0.496
	GA + AA	6.05 (5.31)			6.67 (4.04)		
HOMA-IS	GG	0.01 (0.02)	−0.001 (−0.004, 0.003)	0.681	0.01 (0.01)	0.001 (−0.003, 0.004)	0.623
	GA + AA	0.01 (0.01)			0.01 (0.01)		
Cholesterol (mg/dl)	GG	164.74 (46.57)	−11.075 (−28.379, 6.230)	0.208	158.96 (33.16)	−8.095 (−23.264, 7.074)	0.294
	GA + AA	153.52 (39.61)			153.25 (36.40)		
Triglycerides (mg/dl)	GG	158.26 (69.32)	−26.360 (−62.172, 9.452)	0.148	105.16 (41.14)	0.337 (−19.156, 19.831)	0.973
	GA + AA	131.67 (86.85)			112.76 (48.02)		
HDL (mg/dl)	GG	27.43 (6.83)	1.274 (−2.235, 4.773)	0.474	31.52 (7.03)	−0.955 (−4.517, 2.606)	0.597
	GA + AA	28.71 (8.18)			30.25 (8.31)		
LDL (mg/dl)	GG	103.56 (40.53)	−9.178 (−24.174, 5.818)	0.229	91.64 (31.62)	−11.629 (−24.169, 0.912)	0.069
	GA + AA	94.10 (36.30)			86.06 (31.38)		
VLDL (mg/dl)	GG	31.51 (13.95)	−4.979 (−12.098, 2.140)	0.169	20.88 (8.26)	0.444 (−3.377, 4.264)	0.819
	GA + AA	26.49 (17.17)			22.64 (9.34)		
CHR	GG	6.18 (1.78)	−0.486 (−1.302, 0.33)	0.241	5.54 (2.37)	−0.389 (−1.107, 0.330)	0.287
	GA + AA	5.69 (1.94)			5.33 (1.57)		
CRI	GG	6.20 (1.79)	−0.579 (−1.313, 0.155)	0.121	5.28 (1.63)	−0.149 (−0.768, 0.470)	0.635
	GA + AA	5.62 (1.72)			5.29 (1.47)		
AI	GG	3.84 (1.31)	−0.429 (−0.949, 0.090)	0.105	3.06 (1.22)	−0.352 (−0.791, 0.087)	0. 115
	GA + AA	3.41 (1.27)			2.94 (0.48)		
TG/HDL-C	GG	6.09 (2.93)	−1.056 (−2.788, 0.677)	0.231	3.63 (2.23)	0.096 (0.763, 0.955)	0.826
	GA + AA	5.03 (4.20)			4.00 (2.02)		

Association of the variant rs2815752 with metabolic traits was assessed by linear regression using dominant model of inheritance. The effect sizes (*β*) with 95% confidence intervals and *p*-values were provided after adjustment for age and BMI.

AI: Atherogenic Index; BP: blood pressure; CHR: cholesterol HDL ratio; CI: confidence interval; CRI: Coronary Risk Index; FBG: fasting blood glucose; HDL: high-density lipoprotein; HOMA-IR: homeostasis model assessment of insulin resistance; HOMA-IS: homeostasis model assessment of insulin sensitivity; LAP: lipid accumulation product; LDL: low-density lipoprotein; SD: standard deviation; TG/HDL-C: triglyceride-to-HDL-C ratio; TyG: product of triglyceride and glucose; VAI: visceral adiposity index; VLDL: very-low-density lipoprotein.

**Table 5.** Assessment of the association of rs2815752 variant with eating behaviour in gender-stratified obese/control pairs using a dominant model of inheritance.

Categorical traits	Response	Males					Females			
		Genotype		Adjusted		*p-*value	Genotype		Adjusted	
		GG	GA + AA	OR (95% CI)	*p-*value	(corrected)	GG	GA + AA	OR (95% CI)	*p-*value
Eating pattern	Random	19 (82.6%)	106 (55.5%)	0.25 (0.08, 0.77)	0.016	0.048^a^	13 (52.0%)	89 (59.7%)	1.16 (0.48, 2.79)	0.742
	Specific	4 (17.4%)	85 (44.5%)				12 (48.0%)	60 (40.3%)		
Diet unconsciousness	Yes	18 (18.3%)	145 (75.9%)	0.88 (0.31, 2.51)	0.807	0.807	15 (60.0%)	103 (69.1%)	1.44 (0.59, 3.52)	0.420
	No	5 (21.7%)	46 (24.1%)				10 (40.0%)	46 (30.9%)		
TFDF	High	10 (43.5%)	66 (34.6%)	0.69 (0.27, 1.71)	0.418	0.627	7 (28.0%)	32 (21.5%)	0.48 (0.17, 1.33)	0.159
	Mod–Low	13 (56.5%)	125 (65.4%)				18 (72.0%)	117 (78.5%)		

Categorical traits were assessed using logistic regression using dominant model. Parameters were adjusted for both age and BMI.

^a^ An adjusted and corrected *p*-value <0.05 was considered statistically significant.

Mod–Low: moderate to low; TFDF: tendency towards fat-dense food.

## Discussion

We found that there was a gender-specific association of the rs2815752 with the obese phenotype (but not with overweight) and some obesity-related anomalous anthropometric traits including weight, BMI, waist circumference, hip circumference, and abdominal and supra-iliac skinfold thicknesses in Pakistani females according to a dominant mode of inheritance. Accordingly, the frequency of the rs2815752 genotypes differed between obese and normal-weight females, with a higher frequency of risk allele (A)-carrying genotypes (GA, AA) and lower frequency of GG genotype in cases as compared to controls. This suggests that the variant may be associated with the extreme phenotype (obesity) but not with the mild phenotype (overweight) in females of our population. Similar to our findings, Rukh et al. also reported a significant association of the variant rs2815752 with obesity (according to additive model adjusted for age and gender) but not with overweight (20) in a Swedish population. In addition, Mägi et al. also reported a strong association of the rs2815752 with higher grade of obesity (BMI ≥ 35.0 kg/m^2^) in Europeans with respect to additive, dominant, and recessive models adjusted for age and gender, but they did not calculate *h* index for indicating the appropriate mode of inheritance (21). A possible explanation for the observed association of the rs2815752 with obesity but not with overweight could be that the genetic variants that confer susceptibility to weight gain tend to concentrate in extreme phenotypes (BMI ≥ 30) as compared to mild phenotype (BMI ≥ 25) (22). Contrary to our findings, a study on Germans showed no association of the variant rs2815752 with obesity in gender-adjusted overall as well as gender-specific separate analyses considering the additive model (23). Furthermore, a study on Chinese children revealed no association of the variant with obesity according to a gender-adjusted log-additive model (24). However, a study conducted on Mexican children found a nominal association of the variant with obesity risk, employing an additive model adjusted for age and gender (25). In the current study, the association of the *NEGR1* rs2815752 with many obesity-related anthropometric parameters including weight, BMI, WC, HC, abdominal SFT, and supra-iliac SFT but not with other anthropometric traits in obese females indicates that this variant contributes to obesity by higher fat deposition in only the waist, hip, and abdomen.

The association of the variant rs2815752 with BMI, WC, and HC has also been reported by Rukh et al. in a Swedish population (20). Likewise, a study on south Brazilian children also showed a significant association of the variant rs2815752 with BMI and sum of skin-folds (26). In contrast, Bauer et al. found no significant association of the *NEGR1* rs2815752 with adiposity measures in Dutch females (27). In addition, there was no association of the variant rs2815752 with birth weight and body fat distribution in a meta-analysis conducted by Kilpeläinen et al. (28).

In some of our previous studies, we also reported gender-specific association of the other variants such as MC4R rs17782313, FTO rs9939609, and LEP rs7799039 with overweight/obesity and related anthropometric traits in Pakistani females (29–31). In comparison to these previously reported associations, the association of *NEGR1* rs2815752 observed in the current study has a higher impact (effect size) but less strength. As mentioned before, this variant has also been reported as having one of the biggest effect sizes on BMI values in GWAS conducted on Caucasians (8–10). Pakistan is one of the countries that has entered stage 1 of the obesity transition characterised by a higher prevalence of obesity in women than in men (32). In this context, the observed associations of genetic variants with obesity and obesity-related anthropometric traits in females of our population indicates the existence of sexual dimorphism in genetic susceptibility to obesity and may partly explain the widening gender gap in excess weight as 10% more women gain weight than men in Pakistan (18). Moreover, gene–environment interactions play an important role in the determination of complex traits like obesity. Aberrant lifestyle may be more common in women as compared to men in Pakistan (33). Thus, the interaction between the genetic variants and aberrant lifestyle may possibly make Pakistani females more prone to gain weight. The strategy of stratified analysis can be more powerful than a combined analysis when a variant is restricted to a subgroup or when the extreme difference of a variant’s effect is present in two genders (34,35). GWAS have also revealed sexual dimorphic effects in genetic loci for adiposity-related phenotypes, such as waist circumference and waist-to-hip ratio with stronger effects in women (36).

Obesity is a major risk factor for a host of metabolic disturbances, predominantly in lipid and glucose metabolism. Epidemiological evidence indicates that adiposity is associated with aberrant lipid profiles and biomarkers of glucose metabolism (37,38). On the same lines, all the metabolic variables of the current study appeared significantly aberrant in cases as compared to controls. However, there was no association of the *NEGR1* rs2815752 with any of the metabolic parameters in the current study. In a similar manner, a study by Haupt and colleagues also showed no association of this variant with biomarkers of glucose metabolism such as fasting glucose and insulin sensitivity (39). Moreover, Fall et al. in a study on Swedish non-diabetic old men also reported no association of the rs2815752 with insulin sensitivity (40). In addition to biomarkers of glucose metabolism, we also sought the association of the variant rs2815752 with parameters of lipid profile (total cholesterol, TG, HDL-C, LDL-C, VLDL-C, CHR, and TG to HDL-C ratio) and other metabolic variables including VAI, LAP, TyG index, CRI, AI, systolic blood pressure, and diastolic blood pressure, which have never been investigated before. The lack of association between the rs2815752 and metabolic traits in the current study suggests that the variant rs2815752 may not have a direct impact on these metabolic parameters, but it may influence these parameters indirectly via influencing BMI.

We found no association of the *NEGR1* rs2815752 with aberrant eating behaviours such as tendency towards fat-dense food and diet unconsciousness. The lack of association between the *NEGR1* rs2815752 and eating behaviours may be due to self-reporting bias (under-reporting) of the study participants as relevant data were collected by means of a questionnaire. However, there was a weak (*p* = 0.048) negative (protective) association of the variant rs2815752 with random eating timings in males only. In contrast, Micali et al. reported a nominal (*p* = 0.047) positive association of the rs2815752 with binge-eating (episodes of overeating with loss of control occurring on average once a week over 3 months and accompanied by distress) in male adolescents but not in females (41).

The strengths and the limitations of the current study can be indicated at this point. Our study was based on a case-control design in which cases and controls were matched for age and gender. Many of the obesity-related parameters investigated in our study have not been studied before in relation to the *NEGR1* rs2815752. The study employed multiple genetic models for seeking association of the rs2815752 with obesity via regression analysis. The study also applied the calculation of *h* index in case of getting association in more than one genetic model for an indication of the appropriate mode of inheritance. Analyses were adjusted for covariates such as age and/or BMI depending upon the situation. In addition, the study also included corrections for multiple comparisons in order to avoid false-positive results. The limitations of the current study include the likelihood of self-reporting bias in case of behavioural data that were self-reported by the study participants via a questionnaire. In addition, the sample size was considerably reduced after the gender-based stratification, resulting in decreased statistical power (reduced number of participants in the subgroup by sex). Thus, the findings may be corroborated by further investigations of this association in a considerably larger sample of Pakistani females.

In conclusion, our study revealed a gender-specific association of the *NEGR1* rs2815752 variant with obese phenotype and some related anthropometric traits such as weight, BMI, WC, HC, abdominal SFT, and supra-iliac SFT in females of the Pakistani population. On the other hand, there was no association of the rs2815752 with obesity-related metabolic traits and eating behaviour.

## Supplementary Material

Supplemental Material
