# Supplementary material for: Association of the NEGR1 rs2815752 with obesity and related traits in Pakistani females
Source: Ups J Med Sci. 2020 May 18;125(3):226–34. doi: 10.1080/03009734.2020.1756996 (PMC7875551; doi:10.1080/03009734.2020.1756996)
Supplement: Supplemental Material [file IUPS_A_1756996_SM5830.docx]

**Supplementary Tables File (Manuscript: UJMS-2019-0257.R2)**

| **Table 1** Comparison of continuous variables between overall cases (OW+OB) and normal weight controls | | | | | | | | | |  |
| --- | --- | --- | --- | --- | --- | --- | --- | --- | --- | --- |
| **Continuous variables** | **All (males and females)**  *N* = 612 | | | **Males**  *N* = 336 | | | **Females**  *N* = 276 | | | |
|  |  | | |  | | |  | | | |
|  | **OW+OB**  *N* = 306 | **Controls**  *N* = 306 | ***P-*value** | **OW+OB**  *N* = 168 | **Controls**  *N =* 168 | ***P-*value** | **OW+OB**  *N =* 138 | **Controls**  *N =* 138 | ***P-*value** | |
| **Anthropometric parameters** | | | | | | | | | | |
|  |  |  |  |  |  |  |  |  |  | |
| **Weight (Kg)** | 445.80 | 167.20 | **<0.001*** | 203.93 | 73.07 | **<0.001*** | 248.66 | 88.34 | **<0.001*** | |
| **Height (cm)** | 301.14 | 311.86 | 0.453 | 129.32 | 147.68 | 0.056 | 167.77 | 169.23 | 0.890 | |
| **BMI (Kg/m^2^)** | 459.46 | 153.54 | **<0.001*** | 207.46 | 69.54 | **<0.001*** | 252.50 | 84.50 | **<0.001*** | |
| **WC (cm)** | 449.83 | 163.17 | **<0.001*** | 202.17 | 74.83 | **<0.001*** | 248.56 | 88.44 | **<0.001*** | |
| **HC (cm)** | 146.14 | 166.86 | **<0.001*** | 202.58 | 74.42 | **<0.001*** | 244.34 | 92.16 | **<0.001*** | |
| **WHR** | 410.81 | 202.19 | **<0.001*** | 188.01 | 89.99 | **<0.001*** | 224.84 | 112.16 | **<0.001*** | |
| **Biceps SFT (mm)** | 414.30 | 198.70 | **<0.001*** | 190.34 | 86.66 | **<0.001*** | 233.67 | 103.33 | **<0.001*** | |
| **Triceps SFT (mm)** | 436.04 | 176.96 | **<0.001*** | 194.15 | 82.85 | **<0.001*** | 243.31 | 93.69 | **<0.001*** | |
| **Abdominal SFT (mm)** | 432.75 | 180.25 | **<0.001*** | 194.62 | 82.38 | **<0.001*** | 239.34 | 97.66 | **<0.001*** | |
| **Supra-iliac SFT (mm)** | 427.17 | 185.83 | **<0.001*** | 190.29 | 86.71 | **<0.001*** | 242.48 | 94.52 | **<0.001*** | |
| **Thigh SFT (mm)** | 437.17 | 175.83 | **<0.001*** | 195.22 | 81.78 | **<0.001*** | 243.89 | 93.11 | **<0.001*** | |
| **Sub-scapular SFT (mm)** | 430.26 | 181.33 | **<0.001*** | 198.16 | 78.84 | **<0.001*** | 233.66 | 101.95 | **<0.001*** | |
| **% Body fat** | 442.22 | 170.78 | **<0.001*** | 195.82 | 81.18 | **<0.001*** | 247.19 | 89.81 | **<0.001*** | |
| **Metabolic parameters** | | | | | | | | | | |
| **Systolic BP (mmHg)** | 343.71 | 269.29 | **<0.001*** | 165.13 | 111.87 | **<0.001*** | 178.43 | 158.57 | **0.052*** | |
| **Diastolic BP (mmHg)** | 344.25 | 268.75 | **<0.001*** | 152.89 | 124.11 | **0.002*** | 192.01 | 144.99 | **<0.001*** | |
| **VAI (mmol L-1)** | 352.83 | 260.17 | **<0.001*** | 195.11 | 141.89 | **<0.001*** | 158.89 | 118.11 | **<0.001*** | |
| **LAP (mmol L-1)** | 428.87 | 184..13 | **<0.001*** | 237.72 | 99.28 | **<0.001*** | 191.29 | 85.71 | **<0.001*** | |
| **TyG index** | 363.06 | 249.94 | **<0.001*** | 204.41 | 132.59 | **<0.001*** | 158.64 | 118.36 | **<0.001*** | |
| **FBG (mg/dL)** | 334.92 | 278.08 | **<0.001*** | 153.49 | 123.51 | **0.002*** | 182.44 | 154.56 | **0.009*** | |
| **Insulin (µl U/mL)** | 372..00 | 241.00 | **<0.001*** | 155.06 | 121.94 | **0.001*** | 217.29 | 119.71 | **<0.001*** | |
| **HOMA-IR** | 374.31 | 238.69 | **<0.001*** | 157.30 | 119.70 | **<0.001*** | 217.75 | 119.25 | **<0.001*** | |
| **HOMA-IS** | 238.69 | 374.31 | **<0.001*** | 119.25 | 217.75 | **<0.001*** | 119.74 | 157.26 | **<0.001*** | |
| **Cholesterol (mg/dL)** | 333.31 | 279.69 | **<0.001*** | 143.05 | 133.95 | 0.343 | 190.57 | 146.43 | **<0.001*** | |
| **Triglycerides (mg/dL)** | 357.87 | 255.13 | **<0.001*** | 155.37 | 121.63 | **<0.001*** | 203.10 | 133.90 | **<0.001*** | |
| **HDL-C (mg/dL)** | 316.98 | 296.02 | 0.142 | 134.79 | 142.21 | 0.440 | 183.15 | 153.85 | **0.006*** | |
| **LDL-C (mg/dL)** | 366.06 | 246.94 | **<0.001*** | 155.25 | 121.75 | **<0.001*** | 210.98 | 126.03 | **<0.001*** | |
| **VLDL-C (mg/dL)** | 354.62 | 258.38 | **<0.001*** | 153.98 | 123.02 | **0.001*** | 201.35 | 135.65 | **<0.001*** | |
| **CHR** | 317.88 | 295.12 | 0.111 | 143.68 | 133.32 | 0.280 | 174.25 | 162.75 | 0.278 | |
| **CRI** | 317.92 | 295.08 | 0.110 | 174.14 | 162.86 | 0.287 | 143.73 | 133.27 | 0.276 | |
| **AI** | 354.13 | 258.87 | **<0.001*** | 198.25 | 138.75 | **<0.001*** | 156.78 | 120.22 | **<0.001*** | |
| **TG/HDL-C** | 345.27 | 267.73 | **<0.001*** | 192.46 | 144.54 | **<0.001*** | 153.71 | 123.29 | **0.002*** | |
| Continuous variables are expressed in mean ranks and compared by Mann–Whitney *U* test between cases and controls. Abbreviations; OW+OB: overweight and obese cases, BMI: body mass index, WC: waist circumference, HC: hip circumference, WHR: waist-to-hip ratio, SFT: skin fold thickness, BP: blood pressure, VAI: visceral adiposity index, LAP: lipid accumulation product, TyG: product of triglyceride and glucose, FBG: fasting blood glucose, HOMA-IR: homeostasis model assessment of insulin resistance, HDL-C: high density lipoprotein cholesterol, LDL-C: low density lipoprotein cholesterol, VLDL-C: very low density lipoprotein cholesterol, CHR: cholesterol HDL-C ratio, CRI: Coronary Risk Index, AI: Atherogenic Index, TG/HDL-C: triglyceride-to-HDL-C ratio.*A *P-*value <0.05 was considered significant and shown in bold. | | | | | | | | | | |

| **Table 2** Comparison of categorical variables between overall cases (OW+OB) and normal weight controls | | | | | | | | | | |
| --- | --- | --- | --- | --- | --- | --- | --- | --- | --- | --- |
| **Categorical variables** | **Response** | **All (males & females)**  *N* = 612 | | | **Males**  *N* = 336 | | | **Females**  *N* = 276 | | |
|  |  | **OB+OW**  *N* = 306 | **Controls**  *N* = 306 | ***P-*value** | **OB+OW**  *N* = 168 | **Controls**  *N* = 168 | ***P-*value** | **OB+OW**  *N* = 138 | **Controls**  *N* = 138 | ***P-*value** |
| **Demographic characteristics** | | | | | | | | | | |
|  | | | | | | | | | | |
| **Parental consanguinity ^a^** | Yes  No | 74 (24.2%)  232 (75.8%) | 67 (4.9%)  239 (78.1%) | 0.565 | 45 (26.8%)  123 (73.2%) | 38 (22.6%)  130 (77.4%) | 0.448 | 29 (21.0%)  109 (79.0%) | 29 (21.0%)  109 (79.0%) | 1.00 |
| **Family history of obesity ^b^** | Yes  No | 185 (60.5%)  121 (39.5%) | 105 (34.3%)  201 (55.7%) | **<0.001*** | 89 (53.0%)  79 (47.0%) | 50 (29.8%)  118 (70.2%) | **<0.001*** | 96 (69.6%)  42 (30.4%) | 55 (39.9%)  83 (60.1%) | **<0.001*** |
| **Eating behavior** | | | | | | | | | | |
| **Eating timing** | Random  Specific | 200 (65.4%)  106 (34.6%) | 163 (53.3%)  143 (46.7%) | **0.003*** | 111 (66.1%)  57 (33.9%) | 86 (51.2%)  82 (48.8%) | **0.008*** | 89 (64.5%)  49 (35.5%) | 77 (55.8%)  61 (44.2%) | 0.176 |
| **Diet unconsciousness** | Yes  No | 226 (73.9%)  80 (26.1%) | 210 (68.6%)  96 (31.4%) | 0.152 | 134 (79.8%)  31 (20.2%) | 115 (68.5%)  53 (31.5%) | **0.025*** | 92 (66.7%)  46 (33.3%) | 95 (68.8%)  43 (31.2%) | 0.797 |
| **TFDF** | High  Moderate-low | 113 (36.9%)  193 (63.0%) | 59 (19.3%)  247 (80.7%) | **<0.001*** | 68 (40.5%)  100 (59.5%) | 38 (22.6%)  130 (77.3%) | **0.002*** | 45 (32.6%)  93 (67.4%) | 21 (15.2%)  117 (84.6%) | **0.003*** |
| **Ethnicity** | | | | | | | | | | |
| **Urdu speaking**  **Punjabi**  **Sindhi**  **Pashtun**  **Balochi**  **Other** | | 172 (56.2%)  55 (18.0%)  23 (7.5%)  34 (11.1%)  8 (2.6%)  14 (4.6%) | 169 (55.2%)  56 (18.3%)  24 (7.8%)  30 (9.8%)  5 (1.6%)  22 (7.2%) | **-** | 94 (56.0%)  29 (17.3%)  13 (7.7%)  20 (11.9%)  3 (1.8%)  9 (5.4%) | 81 (48.2%)  18 (10.7%)  21 (12.5%)  26 (15.5%)  4 (2.4%) 18 (10.7%) | **-** | 78 (56.5%)  26 (18.8%)  10 (7.2%)  14 (10.1%)  5 (3.6%)  5 (3.6%) | 88 (63.8%)  38 (27.5%)  3 (2.2%)  4 (2.9%)  1 (0.7%)  4 (2.9%) | **-** |
| Categorical variables are represented as counts with percentages in parenthesis. Demographic characteristics and parameters of eating behavior are compared between cases and controls by Fisher’s exact test. ^a^ Parental consanguinity was taken as marriage with a first cousin. ^b^ Family history of obesity was taken as presence or absence of obesity in parents and siblings. Abbreviations; OW+OB: overweight and obese cases, TFDF, tendency toward fat dense food. *A *P*-value <0.05 was considered significant | | | | | | | | | | |

| **Table 3** Comparison of continuous variables between obese cases (BMI ≥ 30 kg/m^2^) and normal weight controls | | | | | | | | | |  |
| --- | --- | --- | --- | --- | --- | --- | --- | --- | --- | --- |
| **Continuous study variables** | **All (males and females)**  *N* = 388 | | | **Males**  *N* = 214 | | | **Females**  *N* = 174 | | | |
|  |  | | |  | | |  | | | |
|  | **Obese**  *N* = 194 | **Controls**  *N* = 194 | ***P-*value** | **Obese**  *N* = 107 | **Controls**  *N =* 107 | ***P-*value** | **Obese**  *N =* 87 | **Controls**  *N =* 87 | ***P-*value** | |
| **Anthropometric parameters** | | | | | | | | | | |
|  |  |  |  |  |  |  |  |  |  | |
| **Weight (kg)** | 289.47 | 99.53 | **<0.001*** | 159.81 | 55.19 | **<0.001*** | 130.97 | 44.03 | **<0.001*** | |
| **Height (cm)** | 187.90 | 201.10 | 0.246 | 103.50 | 111.50 | 0.346 | 81.13 | 93.87 | 0.095 | |
| **BMI (kg/m^2^)** | 291.50 | 97.50 | **<0.001*** | 161.00 | 54.00 | **<0.001*** | 131.00 | 44.00 | **<0.001*** | |
| **WC (cm)** | 290.48 | 98.52 | **<0.001*** | 160.32 | 54.68 | **<0.001*** | 130.74 | 44.26 | **<0.001*** | |
| **HC (cm)** | 289.55 | 99.45 | **<0.001*** | 159.63 | 55.37 | **<0.001*** | 130.59 | 44.41 | **<0.001*** | |
| **WHR** | 263.97 | 125.03 | **<0.001*** | 144.01 | 70.99 | **<0.001*** | 121.29 | 53.71 | **<0.001*** | |
| **Biceps SFT (mm)** | 270.25 | 118.75 | **<0.001*** | 151.46 | 63.64 | **<0.001*** | 125.05 | 49.95 | **<0.001*** | |
| **Triceps SFT (mm)** | 283.99 | 105.01 | **<0.001*** | 158.45 | 56.55 | **<0.001*** | 126.32 | 49.95 | **<0.001*** | |
| **Abdominal SFT (mm)** | 281.90 | 107.10 | **<0.001*** | 155.86 | 55.14 | **<0.001*** | 127.02 | 47.98 | **<0.001*** | |
| **Supra-iliac SFT (mm)** | 277.33 | 111.67 | **<0.001*** | 157.73 | 57.27 | **<0.001*** | 122.57 | 52.83 | **<0.001*** | |
| **Thigh SFT (mm)** | 287.16 | 101.84 | **<0.001*** | 159.88 | 55.12 | **<0.001*** | 126.76 | 48.24 | **<0.001*** | |
| **Sub-scapular SFT (mm)** | 279.64 | 107.91 | **<0.001*** | 155.33 | 60.20 | **<0.001*** | 127.52 | 47.48 | **<0.001*** | |
| **% Body fat** | 288.09 | 100.91 | **<0.001*** | 159.66 | 55.34 | **<0.001*** | 129.53 | 45.47 | **<0.001*** | |
| **Metabolic parameters** | | | | | | | | | | |
| **Systolic BP (mmHg)** | 227.54 | 161.46 | **<0.001*** | 117.30 | 97.70 | **0.016*** | 110.64 | 64.54 | **<0.001*** | |
| **Diastolic BP (mmHg)** | 223.47 | 155.53 | **<0.001*** | 121.20 | 93.80 | **0.001*** | 102.77 | 72.23 | **<0.001*** | |
| **VAI (mmol L-1)** | 225.97 | 163.03 | **<0.001*** | 124.03 | 90.97 | **<0.001*** | 103.26 | 71.74 | **<0.001*** | |
| **LAP (mmol L-1)** | 278.04 | 110.96 | **<0.001*** | 153.83 | 61.17 | **<0.001*** | 124.68 | 50.32 | **<0.001*** | |
| **TyG index** | 232.18 | 156.82 | **<0.001*** | 129.08 | 85.92 | **<0.001*** | 103.65 | 71.35 | **<0.001*** | |
| **FBG (mg/dl)** | 216.07 | 172.93 | **0.003*** | 116.98 | 98.11 | **0.026*** | 100.30 | 74.70 | **0.001*** | |
| **Insulin (µl U/ml)** | 243.75 | 145.25 | **<0.001*** | 140.77 | 74.23 | **<0.001*** | 103.89 | 71.11 | **<0.001*** | |
| **HOMA-IR** | 245.91 | 143.09 | **<0.001*** | 141.45 | 73.55 | **<0.001*** | 109.01 | 70.85 | **<0.001*** | |
| **HOMA-IS** | 143.09 | 245.91 | **<0.001*** | 73.55 | 141.45 | **<0.001*** | 69.34 | 105.66 | **<0.001*** | |
| **Cholesterol (mg/dL)** | 211.32 | 177.68 | **0.003*** | 120.72 | 94.28 | **0.002*** | 90.26 | 88.77 | 0.368 | |
| **Triglycerides (mg/dL)** | 227.07 | 161.93 | **<0.001*** | 127.76 | 87.24 | **<0.001*** | 100.22 | 74.48 | **0.001*** | |
| **HDL-C (mg/dL)** | 193.34 | 195.66 | 0.839 | 111.72 | 103.28 | 0.319 | 81.96 | 93.04 | 0.147 | |
| **LDL-C (mg/dL)** | 234.43 | 154.57 | **<0.001*** | 134.49 | 80.51 | **<0.001*** | 100.38 | 74.62 | **0.001*** | |
| **VLDL-C (mg/dL)** | 224.78 | 164.22 | **<0.001*** | 126.18 | 88.82 | **<0.001*** | 99.19 | 75.81 | **<0.001*** | |
| **CHR** | 210.83 | 178.17 | **0.004*** | 115.56 | 99.44 | 0.057 | 95.74 | 79.26 | **0.031*** | |
| **CRI** | 210.77 | 178.23 | **0.004*** | 115.01 | 99.99 | 0.076 | 96.07 | 78.93 | **0.025*** | |
| **AI** | 232.70 | 156.30 | **<0.001*** | 130.06 | 84.94 | **<0.001*** | 103.67 | 71.33 | **<0.001*** | |
| **TG/HDL-C** | 223.23 | 165.77 | **<0.001*** | 122.66 | 92.34 | **<0.001*** | 101.25 | 73.75 | **<0.001*** | |
| Continuous study variables are expressed in mean ranks and compared by Mann–Whitney U test between cases and controls. Abbreviations; OW+OB: overweight and obese, BMI: body mass index, WC: waist circumference, HC: hip circumference, WHR: waist-to-hip ratio, SFT: skin fold thickness, BP: blood pressure, VAI: visceral adiposity index, LAP: lipid accumulation product, TyG: product of triglyceride and glucose, FBG: fasting blood glucose, HOMA-IR: homeostasis model assessment of insulin resistance, HDL: high density lipoprotein, LDL: low density lipoprotein, VLDL: very low density lipoprotein, CHR: cholesterol HDL ratio, CRI: Coronary Risk Index, AI: Atherogenic Index, TG/HDL-C: triglyceride-to-HDL-C ratio.*A *P-*value <0.05 was considered significant. | | | | | | | | | | |

| **Table 4** Comparison of categorical study variables between obese cases (BMI ≥ 30 kg/m^2^) and normal weight controls | | | | | | | | | | |
| --- | --- | --- | --- | --- | --- | --- | --- | --- | --- | --- |
| **Categorical study variables** | **Response** | **All (males & females)**  *N* = 388 | | | **Males**  *N* = 214 | | | **Females**  *N* = 174 | | |
|  |  | **Obese**  *N* = 194 | **Controls**  *N* = 194 | ***P-*value** | **Obese**  *N* = 107 | **Controls**  *N* = 107 | ***P-*value** | **Obese**  *N* = 87 | **Controls**  *N* = 87 | ***P-*value** |
| **Demographic characteristics** | | | | | | | | | | |
|  | | | | | | | | | | |
| **Parental consanguinity ^a^** | Yes  No | 46 (23.7%)  148 (76.3%) | 41 (21.1%)  153 (78.9%) | 0.626 | 27 (25.2%)  80 (74.8%) | 26 (24.3%)  81 (75.7%) | 1.000 | 19 (21.8%)  68 (78.2%) | 15 (17.2%)  72 (82.8%) | 0.567 |
| **Family history of obesity ^b^** | Yes  No | 100 (55.5%)  94 (48.5%) | 75 (39.3)  119 (60.7%) | **0.014*** | 54 (50.5%)  53 (49.5%) | 41 (38.3%)  66 (61.7%) | 0.098 | 46 (52.9%)  41 (47.1%) | 34 (39.1%)  53 (60.9%) | 0.094 |
| **Eating behavior** | | | | | | | | | | |
| **Eating timings** | Random  Specific | 131 (67.4%)  63 (32.5%) | 96 (49.5%)  98 (50.5%) | **<0.001*** | 71 (66.4%)  36 (33.6%) | 54 (50.5%)  53 (49.5%) | **0.026*** | 60 (69.0%)  27 (31.0%) | 42 (48.3%)  45 (51.7%) | **0.009*** |
| **Diet unconsciousness** | Yes  No | 148 (76.3%)  46 (23.7%) | 133 (68.6%)  61 (31.4%) | 0.112 | 88 (82.2%)  19 (17.8%) | 75 (70.1%)  32 (29.9%) | 0.054 | 60 (69.0%)  27 (31.0%) | 58 (66.7%)  29 (33.3%) | 0.871 |
| **TFDF ^c^** | High  Moderate-low | 77 (39.7%)  117 (60.3%) | 38 (19.6%)  156 (80.4%) | **<0.001*** | 49 (45.8%)  58 (54.2%) | 27(25.2%)  80 (74.8%) | **0.003*** | 28 (32.2%)  59 (67.8%) | 11 (12.6%)  76 (87.4%) | **0.003*** |
| **Ethnicity** | | | | | | | | | | |
| Urdu speaking  Punjabi  Sindhi  Pakhtun  Balochi  Other | | 112 (57.7%)  35 (18.0%)  10 (5.2%)  23 (11.9%)  7 (3.6%)  7 (3.6%) | 106 (54.6%)  37 (19.1%)  16 (8.2%)  20 (10.3%)  3 (1.5%)  12 (6.2%) | **-** | 65 (60.7%)  17 (15.9%)  5 (4.7%)  14 (13.1%)  3 (2.8%)  3 (2.8%) | 54 (50.5%)  11 (10.3%  14 (13.1%)  17 (15.9%)  2 (1.9%)  9 (8.4%) | **-** | 47 (54.0%)  18 (20.7%)  5 (5.7%)  9 (10.3%)  4 (4.6%)  4 (4.6%) | 52 (59.8%)  26 (29.9%)  2 (2.3%)  3 (3.4%)  1 (1.1%)  3 (3.4%) | **-** |
| Categorical variables are represented as counts with percentages in parenthesis. Demographic characteristics and parameters of eating behavior are compared between cases and controls by Fisher’s exact test. ^a^ Parental consanguinity was taken as history of marriage with a first cousin. ^b^ Family history of obesity was taken as presence or absence of obesity in parents and siblings. ^c^Tendency towards fat dense food. *A *P*-value <0.05 was considered significant. | | | | | | | | | | |

| **Table 5** Genotypic frequencies of the *NEGR1* rs2815752 variant among cases (overweight and obese) and controls and assessment of the rs2815752 association with overweight/obesity | | | | | | |
| --- | --- | --- | --- | --- | --- | --- |
| **Genetic**  **Model** | **Genotype** | **Cases**  **Count (%)** | **Controls**  **Count (%)** | **Adjusted OR^a^**  **(95% CI)** | | **Adjusted**  ***P*-value^a^** |
| Co-dominant | GG | 35 (11.44) | 42 (13.73) |  | | - |
|  | GA | 133(43.46) | 132 (43.14) | - | |  |
|  | AA | 138 (45.10) | 132 (43.14) |  | |  |
| Pair-wise  comparison | GG vs. GA  GG vs. AA | - | - | 1.207  (0.724-2.012)  1.278  (0.767-2.128) | | 0.470 |
|  |  |  |  |  |  | 0.347 |
| Dominant | (GA+AA) | 271(88.56) | 264 (86.27) | 1.242 | | 0.378 |
|  | GG | 35 (11.44) | 42 (13.73) | (0.768-2.009) | |  |
| Recessive | AA | 138 (45.10) | 131 (42.81) | 1.104 | | 0.546 |
|  | (GG+GA) | 168 (54.90) | 175 (57.19) | (0.801-1.521) | |  |
| Over-dominant | GA | 133 (43.46) | 133 (43.46) | 0.998 | | 0.990 |
|  | (GG+AA) | 173 (56.54) | 173 (56.54) | (0.724-1.376) | |  |
| **Hardy–Weinberg Equilibrium (HWE):** | | | | | | |
| Cases |  |  |  |  | 0.729 | |
| Controls |  |  |  |  | 0.333 | |
| Data represents genotype counts and percentages in parenthesis in cases and controls. Data also indicate genotypic frequencies in HWE. Associations were tested using chi-square and multinomial regression for co-dominant model and binary logistic regression for other models. ^a^Analysis was also performed with adjustment for age and gender. An adjusted *P*-value <0.05 was considered significant. Abbreviations: GG: wild-type homozygous genotype; GA: heterozygous genotype; AA: mutant homozygous genotype; OR: odds ratio; CI: confidence interval. | | | | | | |
